# EMQN best practice guidelines for molecular genetic testing and reporting of 21-hydroxylase deficiency

**DOI:** 10.1038/s41431-020-0653-5

**Published:** 2020-07-02

**Authors:** Sabina Baumgartner-Parzer, Martina Witsch-Baumgartner, Wolfgang Hoeppner

**Affiliations:** 1 Department of Internal Medicine III, Clinical Division of Endocrinology & Metabolism, Medical University of Vienna, Währinger Gürtel 18-20, A-1090 Vienna, Austria; 2 Department of Medical Genetics, Molecular and Clinical Pharmacology, Division Human Genetics, Medical University of Innsbruck, Peter-Mayr-Straße 1, A-6020 Innsbruck, Austria; 3 Bioglobe GmbH, Grandweg 64, D-22529 Hamburg, Germany

**Keywords:** Adrenal gland diseases, Genetic testing

## Abstract

Molecular genetic testing for congenital adrenal hyperplasia (CAH) due to 21-hydroxylase deficiency (21-OHD) is offered worldwide and is of importance for differential diagnosis, carrier detection and adequate genetic counseling, particularly for family planning. In 2008 the European Molecular Genetics Quality Network (EMQN) for the first time offered a European-wide external quality assessment scheme for CAH (due to 21-OH deficiency). The interest was great and over the last years at about 60 laboratories from Europe, USA and Australia regularly participated in that scheme. These best practice guidelines were drafted on the basis of the extensive knowledge and experience got from those annually organized CAH-schemes. In order to obtain the widest possible consultation with practicing laboratories the draft was therefore circulated twice by EMQN to all laboratories participating in the EQA-scheme for CAH genotyping and was updated by that input. The present guidelines address quality requirements for diagnostic molecular genetic laboratories, as well as criteria for *CYP21A*2 genotyping (including carrier-testing and prenatal diagnosis). A key aspect of that article is the use of appropriate methodologies (e.g., sequencing methods, MLPA (multiplex ligation dependent probe amplification), mutation specific assays) and respective limitations and analytical accuracy. Moreover, these guidelines focus on classification of variants, and the interpretation and standardization of the reporting of *CYP21A2* genotyping results. In addition, the article provides a comprehensive list of common as well as so far unreported *CYP21A2*-variants.

## Introduction—CAH (21-OH deficiency)

### Best practice guidelines

Congenital adrenal hyperplasia (CAH) is an autosomal recessive disorder, caused in more than 90% of cases by variants of *CYP21A2* impairing the function of 21-hydroxylase [1–4]. Since *CYP21A2* genotyping is of importance for differential diagnosis, carrier detection and adequate genetic counseling, it is performed worldwide and completes 21-OH deficiency newborn screening offered in many countries. Encouraged by the European Molecular Genetics Quality Network (EMQN) and its European-wide external quality assessment scheme for CAH (due to 21-OH deficiency) best practice guidelines were drafted on the basis of the extensive knowledge and experience got from those annually organized CAH-schemes.

In order to obtain the widest possible consultation with practicing laboratories the draft was therefore circulated twice by EMQN to all laboratories participating in the EQA-scheme for CAH genotyping and was updated by that input. Nevertheless, the authors are aware that due to the wide range of 21-OHD phenotypes, the complex genetic background and different legal obligations, it will be difficult to achieve consensus for all issues addressed in this paper.

In deficiency of 21-hydroxylase (21-OHD), a cytochrome P450 enzyme (P450C21) involved in cortisol biosynthesis, 21-hydroxylation is impaired in the zona fasciculata of the adrenal cortex so that 17α-hydroxyprogesterone (17α-OHP) and progesterone are not converted to 11-deoxycortisol and 11-deoxycorticosterone, respectively. Owing to the resulting diminished plasma concentrations of aldosterone and cortisol, ACTH levels increase, resulting in overproduction and accumulation of cortisol precursors (particularly 17α-OHP), which are finally diverted to androsterone and testosterone [5–7]. Variable degrees of prenatal and/or postnatal androgen excess as well as diminished cortisol and aldosterone synthesis reflect the broad clinical range of CAH due to 21-OHD.

From the clinical point of view 21-OHD is categorized into classic (including life threatening salt-wasting as well as simply virilizing), non-classic or so-called cryptic forms of the disease [1–3], the latter often not brought to medical attention. Key features of classic 21-OHD in newborns are ambiguous genitalia in females, neonatal salt loss, failure to thrive and potentially fatal hypovolemia and shock [8, 9]. In the following years classical CAH is characterized by rapid postnatal growth, sexual precocity, different signs of hyperandrogenism, reduced fertility and a variety of additional health problems [10–12].

In contrast to the classical salt-wasting and simple virilizing forms of 21-OHD, the non-classical forms are milder [13, 14], present without adrenal insufficiency and show variable symptoms of postnatal androgen excess (premature pubarche, hirsutism, acne, menstrual abnormalities, unfulfilled pregnancy) or are without clinical symptoms (cryptic CAH). Recent data suggest a reduced quality of life and cognition in CAH patients due to the chronic illness and excess exposure to androgens and corticosteroids [15, 16]. Thus, early diagnosis and adequate treatment is of major importance for mild as well as severe cases of 21-OHD, particularly for children in order to omit unnecessary suffering and to reduce future health problems in adult life [3, 4, 10, 17, 18].

On the basis of neonatal screening programs [8, 9, 19], which are performed in many countries to avoid life threatening salt wasting crises in newborns, the classical forms of CAH are suggested to occur with an incidence of about 1:16,000 in most populations, with a higher incidence reported in Germany (≈1:12,000 for 2005–2007, National Screening Report, www.screening-dgns.de). Non-classic forms of the disease are assumed to be more common [9, 20] and to occur in 0.1–0.2% of the normal Caucasian population. In that context it is of note that recent findings suggest a higher carrier frequency in the general population (of 15% in a Spanish [20] and of 10% in a middle European [21] population) than previously deduced from neonatal screening programs.

Functional impairment of the 21-hydroxylase is caused by variants affecting the function of the *CYP21A2* gene, which is located on chromosome 6p21.33 (Genome assembly GRCh 38). 21-OHD’s broad clinical range reflects the fact that the majority of patients are compound heterozygous for at least two of the more than 200 disease causing *CYP21A2* variants identified so far. In general there is a good phenotype–genotype correlation, the milder variant determining the phenotype [22–24]. Of note, however, recent data suggest that variants in the promoter as well as in the 3′UTR could influence the phenotype [25, 26]. Comprehensive genotyping is therefore of importance for adequate genetic counseling for further pregnancies, the patients’ offspring and family planning.

*CYP21A2* genotyping is error-prone in particular due to the presence of the closely located highly homologous pseudogene *CYP21A1P*, as well as complex duplications, deletions and rearrangements within chromosome 6p21.3. *CYP21A2* genotyping, interpretation of the respective results and adequate genetic counseling of the patient and his/her family members thus require a deep knowledge of *CYP21A2* genetics [27–31].

## Quality requirements for diagnostic molecular genetic laboratories

- Diagnostic *CYP21A2* genotyping should be performed only by accredited laboratories (ISO 15189 or ISO 17025) or laboratories with implemented laboratory quality management systems (equivalent to ISO 15189) and in accordance with local laws and standards.
- Most of the methods used for molecular genetic analyses including sequence analysis, MLPA and Southern Blotting are not CE-IVD certified and therefore diagnostic laboratories should fully validate their analytical procedures before implementing the respective method for patient testing. Nevertheless, it is also necessary for CE-IVD-marked reagents to verify their performance before clinical implementation. For laboratories outside of Europe different regulations and requirements may be relevant and necessary.
- As a minimum, validation should cover an estimate of test accuracy (specificity and sensitivity) and robustness [32]. Test accuracy can be established by analyzing a series of samples of known genotype, and/or by comparison with other laboratories.
- Written Standard Operating Procedures (SOPs) for the methods used are mandatory.
- Laboratories should also refer to and be acquainted with the OECD Guidelines for Quality Assurance of Molecular Genetic Testing, 2007, as well as with EMQN reporting and internal quality control guidelines.
- Annual participation in external quality assessment schemes for *CYP21A2* genotyping (EMQN, UKNEQAS, or similar) is essential.
- A profound knowledge on the molecular background of the disease, the gene structure, and the respective literature is necessary to correctly interprete the results obtained by *CYP21A2* genotyping and to provide adequate genetic counseling.

## Pre-test requirements and criteria for testing

### Test referral, case history, and essential samples

- Most referrals to the genetic laboratory for *CYP21A2*-genotyping come from endocrinologists, pediatricians and obstetricians and the respective reasons are listed in Table 1.

**Table 1:** Reasons for referrals for CYP21A2-genotyping.

**Fig. 1:**
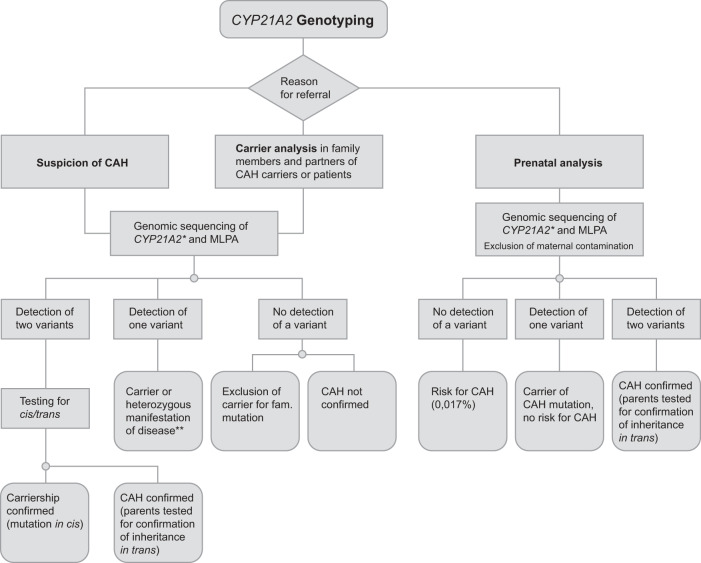
Flow chart of *CYP21A2* genotyping. *Best practice genotyping should be PCR-based sequence analysis along with MLPA as addressed in 6.1. of the manuscript. **Second pathogenic variant in trans not detected.
- It is still controversial whether an ACTH-stimulation test should be performed as the first test for carrier detection in clinically unaffected partners of CAH-patients or suspected carriers. Due to the circumstance that there is an overlap with normal subjects and carriers [33] and that false positive testing has been reported previously [34], 21-OHD genotyping is superior to the stimulation test. However, due to higher costs of the genotyping, the suggested approaches for carrier detection vary and depend on country and region as well as on the respective availability of tetracosactide (active substance of the medicinal product Synacthen^®^).
- Since diffuse virilization or elevated levels of different hormones require the exclusion of a hormone producing neoplasm from the clinical side, laboratories could recommend this in parallel to *CYP21A2* genotyping.
- There is broad agreement that parental samples should be requested together with sampling of the index patient (in case he/she is a child) or when reporting (compare Table 2).

• For both sexes
- Elevated 17α-OHP levels detected by CAH-newborn screening
- Salt loss and failure to thrive
- Precocious puberty
- Accelerated growth in childhood, reduced adult height
- Elevated concentrations of androgens (testosterone), of 17-OHP and of 21- deoxycortisol (basal and after ACTH-stimulation test)
- Transsex or transgender patients before sex reassignment surgery
- Carrier detection in at-risk relatives and in partners of CAH-patients or carriers
- In couples before IVF and ART
- Prenatal diagnosis in at-risk pregnancies
- Adrenal hyperplasia (tumors)
• For females
- Profound clinical symptoms, especially virilization of external genitalia
- Diffuse signs of virilization including hirsutism or acne vulgaris
- Differential diagnosis in patients with PCOS
- Infertility, recurrent miscarriage, abnormal menstrual cycle
• For males
- Swelling of testes reflecting growth of adrenal rests in testicular tissue (TARTs-testicular adrenal rest tumors) [4, 225, 226].
- The type of the test required—confirmation of diagnosis in index patient, prenatal diagnosis, identification of carrier, family analysis (compare Fig. 1)—must be clearly defined by the requesting clinician or geneticist in writing. A proper clinical examination and appropriate biochemical work up is desirable so that a brief clinical description and 17-OHP levels as well as family history are available before diagnostic *CYP21A2* genotyping is performed.

**Table 2:** Analysis of parental samples.

Analysis (CYP21A2 genotyping) of parental samples is necessary in order to determine
- whether heterozygous disease causing variants are in trans or in cis as to calculate the risk of the patient’s children to be carriers and provide adequate genetic counseling for pregnancies in the future
- whether disease causing variants detected in a child are de novo or inherited as to calculate the parents’ risk for further children suffering from CAH and provide adequate genetic counseling
- allocation of different (more than one or two) detected disease causing variants to the different CYP21A2-copies particularly if more than two CYP21A2-copies were detected

### Informed consent and genetic counseling

Policy and procedures concerning initiation of the genotyping, written informed consent, genetic counseling before and after genotyping as well as documentation of genotyping results in different medical records are subject to local practices and national law and have to be handled accordingly. As addressed previously due to the complex genetic background and the wide range of clinical phenotypes *CYP21A2* genotyping and genetic counseling should only be offered by experienced laboratories or institutions with a deep understanding thereof.

## Significance/scope of *CYP21A2* genotyping

### Second-tier confirmatory test

Second-tier confirmatory tests of neonatal CAH screening depend on country and/or region and could be both biochemical and molecular genetic approaches. As reported recently, tandem mass spectrometry has proved to be an excellent second-tier test in newborns with positive CAH screening to confirm elevated 17-OHP levels [35–37]. On the other hand it is an advantage of the molecular genetic screens [38–41] that variants affecting 21-hydroxylase function can be detected in DNA extracted from the same dried blood spots that are used for hormonal neonatal screenings. Because more than 90% of disease-causing alleles carry one out of 10 known variants, most 21-OHD cases could be identified by a screening for those 10 most common variants affecting 21-OH function. A relatively easy to handle “strip-test” [40] covering those most common variants as well as a Real-Time-PCR-based assay to detect copy number variations were recently developed, are commercially available and are used in a limited number of institutions in Europe. Of importance, however, the high carrier frequency reported for Europe [21, 42] in a considerable number of samples requires verification of such results by sequencing the whole gene in order to detect other than the most common and known 10 disease causing variants. In contrast to those commercially available test kits, genotyping particularly including sequencing of the whole gene in combination with MLPA or quantitative PCR (for detection of deletions) is more elaborate, concerning both costs and personnel, but would—due to detection of uncommon and new variants as well as duplications and deletions—spare subsequent genotyping necessary for genetic counseling. The latter aspect of detection of uncommon and new variants (not covered by variant specific assays) is particulary important in less well studied populations and in countries with ethnically diverse populations. In general, all negative variant specific sequencing tests should be followed up by expanded testing particularly if there is a positive screening indication or positive family history. It has to be taken into account, however, that now new techniques such as massive parallel sequencing will allow to screen several CAH genes at once in less time with less costs.

### Exclusion/confirmation of 21-OHD

Comprehensive *CYP21A2* genotyping (not variant specific assays alone) is the best approach to exclude/confirm 21-OHD and 21-OHD-heterozygosity (carrier), because

- particularly mild clinical symptoms as hyperandrogenism, hirsutism, and acne, or infertility [43–47] show overlap with other diseases (e.g., PCOS),
- biochemical parameters (e.g., 17-OHP) should have been measured on certain days of the estrous cycle, which in practice turns out to be difficult. Thus, 17-OHP-levels determined in different laboratories (with blood samples from different days of the estrous cycle) show significant differences hampering interpretation.
- implementation of an ACTH-stimulation test may not be possible in all institutions and depends on availability of Synacthen^®^ (tetracosactide, adrenocorticotropic hormone).

### Family planning—genetic counseling

According to the Endocrine Society Clinical Practice Guidelines [3] genetic counseling should be given to parents at birth of a CAH child and to adolescents at the transition to adult care. To provide correct genetic counseling it should be considered that

- it is only *CYP21A2* genotyping that allows the detection and confirmation of the carrier state in parents with a child suffering from 21-OHD.
- it is of major importance to know whether both parents are carriers or whether a variant affecting 21-OH function in the affected child has occurred de novo.
- it is indispensable to know the nature of the variants affecting function, which determine the type of CAH from which future children could suffer (salt wasting versus simple virilizing versus nonclassical CAH), since this may influence the parents’ decision for future pregnancies.
- the nature of the variants affecting 21-OH function is also required for genetic counseling, because they determine whether prenatal diagnosis and prenatal treatment should be considered in further pregnancies.
- *CYP21A2* genotyping is the best method to detect the carrier state in clinically asymptomatic at risk relatives (family analysis) or partners of CAH-patients and of CAH-carriers.
- in case of in vitro fertilization (IVF): it should be mentioned that it is still a matter of debate whether 21-hydroxylase deficiency genotyping should be considered before IVF and assisted reproductive technology (ART) programs [43, 48, 49].

### Prenatal diagnosis (PND) and prenatal therapy (PNT)

Due to the good genotype/phenotype correlation [1, 2, 22, 23] *CYP21A2* genotyping of the fetus is predictive for his/her clinical outcome. Therefore, several institutions offer *CYP21A2* genotyping in the course of prenatal diagnosis and dexamethasone therapy [50–52]. It is, however, important to mention that prenatal therapy is still considered to be an experimental one [3] due to controversial data particularly concerning treatment outcome and maternal as well as fetal safety [53–55]. Whereas Miller [53] only recently recommended that “fetal endocrine therapy for congenital adrenal hyperplasia should not be done”, due to the Endocrine Society Clinical Practice Guideline [3] prenatal therapy should be pursued through protocols approved by Institutional Review Boards at centers capable of collecting outcome data, and laboratories should seek for cooperation with such centers in order to provide the best counseling for family planning for such patients and to optimize the therapeutic regimen. Nevertheless in those Guidelines [3] the authors state that prenatal treatment of CAH remains controversial and poses unresolved ethical questions.

In general, most often chorionic villus sampling (CVS) is performed at about 10–12 weeks of gestation (or amniocentesis at 14–16 weeks) followed by determination of sex/karyotype by PCR or cytogenetics (often to exclude other chromosomal abnormalities).

By analysis of free fetal DNA from maternal blood nowadays it is possible to perform fetal sex assignment as early as in the sixth/seventh week of gestation [54–57]. The latter technique (NIPD; non-invasive prenatal diagnosis) enables sex determination before the start (7th–8th week of gestation) of a potential therapy and helps to minimize the duration of unnecessary dexamethasone treatment in male or unaffected female pregnancies and has become an integral part of prenatal therapy during the last years [52, 58].

NIPD is a rapidly-expanding technique not only for sex determination, but could also be used for detection of *CYP21A2* variants using massively parallel sequencing (MPS) of cell-free fetal DNA in maternal plasma. Further development of these techniques will provide new possibilities for the diagnosis of monogenic disorders in utero avoiding the complications of invasive testing by chorionic villus sampling [58, 59].

So far PND has been offered to couples for whom it has previously been proven by diagnostic testing that both partners carry severe disease causing *CYP21A2*-variants, which would result in severe virilisation of female external genitalia. By genotyping of the parents de novo variants impairing 21-OH function in previous affected children have been ruled out.

In conclusion the requirements for PND are that

- All steps of PND are performed in accordance with national law and local practice.
- extensive genetic counseling (including information on risks of dexamethasone therapy for mother and child) by a specialist (medical geneticist, clinical specialist, pediatrician, endocrinologist or equivalent) has been performed,
- evidence of written informed consent for PND signed by the respective specialist (who did the pre-test genetic counseling) and the parents is provided with the referral, in accordance with local practices and national law,
- *CYP21A2* genotyping is preceded by fetal sex assignment at the earliest time point possible (6th and 7th week of gestation),
- contamination of the prenatal sample (CVS or amniotic fluid) by maternal tissue is excluded (for example by analysis of polymorphic markers),
- full gene analysis of *CYP21A2* (sequence analysis of all exons and exon/intron boundaries) including copy number detection (MLPA analysis) can be performed, because spontaneous (de novo) variants impairing 21-OH function can occur,
- absence/presence of *CYP21A2* disease causing variants should be confirmed using different techniques (e.g., sequence analysis, MLPA, microsatellite-analysis).
- It has to be kept in mind that non-paternity is a caveat/limitation for test result interpretation and indication for PND. Thus, if possible, parallel testing of parental samples is recommended.

## 21-OH genotyping

### Nomenclature and gene

The nomenclature of the gene, its localization, transcript and protein is based on specifications by the HUGO Gene Nomenclature Committee (HGNC, genenames.org), Online Mendelian Inheritance in Man (OMIM, omim.org) and Locus Reference Genomic (LRG, lrg-sequence.org). Details are given in Table 3.

**Table 3:** Reference sequences and chromosomal localization.

HGNC: CYP21A2, HGNC ID: 2600
Aliases: CYP21, steroid 21-hydoxylase, CYP21B, cytochrome P450, 21-hydroxylase
OMIM Gene *613815
Associated disease: OMIM #201910
Reference sequences
Gene: NG_007941.3
LRG: LRG_829 (based on NG_007941.3)
Transcript: t1 (based on NM_000500.9; ENST00000644719.2)
Protein: p1 (based on NP_000491.4; ENSP00000496625.1)
Consensus Coding Sequence Project (CCDS): 4735.1
Chromosomal localization
Gene map locus 6p21.33
Chromosome 6, NC_000006.12 (32038316..32041670) (built GRCh38:CM000668.2)

### General information on gene, disease causing variants, and genotype–phenotype correlation

#### Gene

The functional *CYP21A2* gene and its highly homologous pseudogene *CYP21A1P* are located in the HLA major histocompatibility complex on chromosome 6p21.33, a highly variable region [60, 61]. Before cloning of the *CYP21A* genes, prenatal diagnosis was performed by HLA-typing. Moreover, particular forms of 21-OH deficiency, e.g., specific variants or constellations such as the p.(Val281Leu) variant c.841G>T or deletions of the pseudogene are associated with certain HLA haplotypes [62].

**Fig. 2:**
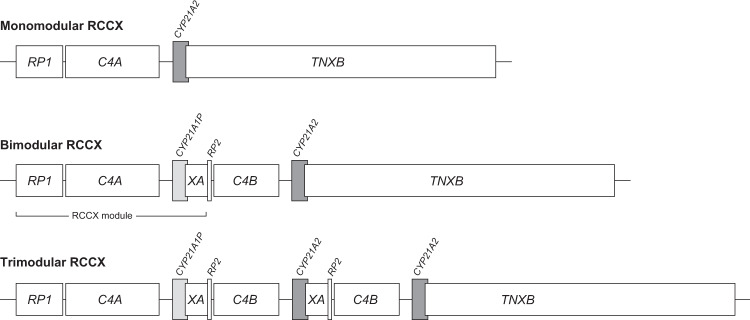
Illustration of a monomodular and a standard bimodular RCCX-unit, composed of the functional *CYP21A*2 gene, the *CYP21A1*P pseudogene and their neighboring genes tenascin *TNXA/B*, complement component 4A/B and the serine/threonine nuclear protein kinase RP. The close proximity of *CYP21A2* and *CYP21A1P* as well as copy number variations for this RCCX- locus could result in a deletion of a 30 kb region (including the *CYP21A2* gene) due to unequal crossing-over (misalignment of a CYP21A1P pseudogene to the functional CYP21A2 gene) between chromosomes with a monomodular and a standard bimodular form.

Both the functional *CYP21A2* gene and the pseudogene form a genetic unit (designated as *RCCX* module) with several neighboring genes including *TNXB*. Copy number variations for this *RCCX* locus have been described, with two modules representing the norm and three or four modules being rare (compare Fig. 2) [63, 64].

#### Disease causing variants

The most frequent variants impairing 21-OH function are the result of recombination and/or gene conversion events between the functional *CYP21A2* gene and its functionally inactive (rendered inactive by variants impairing 21-OH function) pseudogene *CYP21A1P* [1, 2, 65–67]. In a recent review Concolino et al. [68] listed more than 200 CAH causing variants of the *CYP21A2* gene. These include the following most common variants affecting 21-OH function representing about 70–75% of CAH alleles listed in Table 4. In that context it has to be considered that uncommon and new variants (not covered by variant specific assays) could occur with higher frequency in less well studied populations and in countries with ethnically diverse populations. Thus, negative variant specific sequencing tests should be followed up by expanded testing particularly if there is a positive screening indication or positive family history.

**Table 4:** Most common disease causing *CYP21A2* variants (NM_000500.9) detected in Non-Finnish Europeans (Caucasians) in the course of routine *CYP21A2* genotyping performed in the authors’ laboratories.

cDNA level (NM_000500.9)	Predicted protein change (NP_000491.4)	dbSNP	Corresponding exon (NG_007941.3)
c.92C>T	p.(Pro31Leu)	rs9378251	Exon 1
c.293-13C>G	Splicing defect	rs6467	Intron 2
c.332_339del	p.(Gly111Valfs*21)	rs387906510	Exon 3
c.518T>A	p.(Ile173Asn)	rs6475	Exon 4
c.710T>A	p.(Ile237Asn)	rs111647200	Exon 6
c.713T>A	p.(Val238Glu)	rs12530380	Exon 6
c.719T>A	p.(Met240Lys)	rs6476	Exon 6
c.844G>T	p.(Val282Leu)	rs6471	Exon 7
c.923dup	p.(Leu308Phefs*6)	rs267606756	Exon 7
c.955C>T	p.(Gln319*)	rs7755898	Exon 8
c.1069C>T	p.(Arg357Trp)	rs7769409	Exon 8

Variants detected in authors’ departments and so far not published (Table 5) have all been submitted to ClinVar (www.ncbi.nlm.nih.gov/clinvar/). A more comprehensive list of known disease-causing and benign variants is given in Table 6.

**Table 5:** *CYP21A2* variants detected in authors’ institutions (and so far not described in the literature) with ClinVar accession IDs.

Classi-fication	Variant- cDNA level (NM_000500.9)	Predicted protein change (NP_000491.4)	dbSNP	Clin Var accession ID	Corresponding exon (NG_007941.3)
C2	c.-211T>A			Submitted	Pro
C2	c.-210T>C			Submitted	Pro
C1	c.-187A>C			Submitted	Pro
C3	c.-125G>A		rs1377266725	Submitted	5′UTR
C3	c.-121C>T		rs183137942	Submitted	5′UTR
C3	c.50G>T	p.(Arg17Leu)		VCV000800572	Exon 1
C3	c.137C>G	p.(Pro46Arg)		VCV000800573	Exon 1
C1	c.203-18G>C			VCV000800574	Intron 1
C2	c.203-46C>T			VCV000800575	Intron 1
C3	c.268G>T	p.(Ala90Ser)	rs1185350916	VCV000800576	Exon 2
C3	c.292+3A>G		rs752771213	VCV000800577	Intron 2
C2	c.292+37T>A			VCV000800578	Intron 2
C2	c.292+45_292+46insTGT			VCV000800579	Intron 2
C2	c.292+56T>G			VCV000800580	Intron 2
C1	c.292+109C>G			VCV000800581	Intron 2
C2	c.293-136C>T			VCV000800582	Intron 2
C3	c.293-131_290-129dup			VCV000800583	Intron 2
C3	c.293-100_293-99insG			VCV000800584	Intron 2
C1	c.293-130C>T			VCV000800585	Intron 2
C1	c.293-115C>G			VCV000800586	Intron 2
C1	c.293-96G>T			VCV000800587	Intron 2
C1	c.293-95G>C		rs1382005578	VCV000800588	Intron 2
C1	c.293-94T>A			VCV000800589	Intron 2
C1	c.293-91G>A		rs1051507539	VCV000800590	Intron 2
C1	c.293-89A>G			VCV000800591	Intron 2
C1	c.293-88G>A		rs1282239643	VCV000800592	Intron 2
C1	c.293-80G>A		rs79249676	VCV000800593	Intron 2
C1	c.322C>T	p.(Leu108=)		VCV000800611	Exon 3
C1	c.382C>T	p.(Leu128=)		VCV000800612	Exon 3
C1	c.405C>T	p.(Ser135=)		VCV000800613	Exon 3
C1	c.447+38C>T		rs6466	VCV000800614	Intron 3
C1	c.447+39G>A		rs569670804	VCV000800615	Intron 3
C1	c.448-50G>A		rs780875791	VCV000800616	Intron 3
C1	c.448-3C>T			VCV000800617	Intron 3
C3	c.485A>G	p.(Glu162Gly)	rs1229809778	VCV000800618	Exon 4
C3	c.499C>G	p.(Leu167Val)		VCV000800619	Exon 4
C3	c.500T>G	p.(Leu167Arg)	CM071684	VCV000800620	Exon 4
C5	c.509G>A	p.(Cys170Tyr)		VCV000800621	Exon 4
C5	c.525C>A	p.(Tyr174*)		VCV000800622	Exon 4
C3	c.540C>G	p.(Asp180Glu)		VCV000800623	Exon 4
C1	c.550-19C>G			VCV000800624	Intron 4
C4	c.559T>G	p.(Leu187Val)		VCV000800625	Exon 5
C1	c.651+30G>A		rs777741541	VCV000800626	Intron 5
C1	c.651+35A>G		rs12525076	VCV000800627	Intron 5
C1	c.652-5C>T		rs758449746	VCV000800628	Intron 5
C3	c.724C>G	p.(Leu242Val)		VCV000800629	Exon 6
C3	c.738+75C>T		rs1463196531	VCV000800630	Intron 6
C3	c.739-74G>A			VCV000800631	Intron 6
C4	c.754G>A	p.(Gly252Ser)	rs182942340	VCV000800632	Exon 7
C4	c.782T>G	p.(Met261Arg)		VCV000800633	Exon 7
C4	c.782T>C	p.(Met261Tyr)		VCV000800634	Exon 7
C3	c.856G>T	p.(Ala286Ser)		VCV000800594	Exon 7
C3	c.1109G>C	p.(Arg370Pro)		VCV000800595	Exon 8
C3	c.1132G>T	p.(Asp378Tyr)		VCV000800596	Exon 9
C3	c.1170A>T	p.(Gln390His)		VCV000800597	Exon 9
C3	c.1201A>G	p.(Arg401Gly)	rs1451687726	VCV000800598	Exon 9
C1	c.1223-21C>T		rs755724055	VCV000800599	Intron 9
C3	c.1223-3C>G		rs6460	VCV000800600	Intron 9
C5	c.1272C>A	p.(Pro424*)		VCV000800601	Exon 10
C5	c.1291G>A	p.(Gly431Ser)	CD110266	VCV000800602	Exon 10
C3	c.1298C>G	p.(Pro433Arg)		VCV000800603	Exon 10
C1	c.1320C>T	p.(Phe440=)	rs1188690556	VCV000800604	Exon 10
C3	c.1371C>A	p.(Asp457Glu)		VCV000800605	Exon 10
C3	c.1405A>G	p.(Ser469Gly)		VCV000800606	Exon 10
C3	c.1447C>A	p.(Pro483Thr)		VCV000800607	Exon 10
C4	c.1450dup	p.(Arg484Profs*40)		VCV000800608	Exon 10
C1	c.*2G>C		CM1211226	VCV000800609	3′UTR
C1	c.*18C>T			VCV000800610	3′UTR

Classification is done according to ACMG guidelines [98–100], C1 (benign), C2 (likely benign), C3 (uncertain), C4 (likely pathogenic), C5 (definitely pathogenic).

**Table 6:** Listing and classification of known *CYP21A2*-variants (based on NM_000500.9).

Classification	Gene level	Protein level	Id	Region	Phenotype	Reference
C5	c.-126C>T	Transcript. activity ~ 52%	rs191516492	5′UTR	NC	[80]
C5	c.-113G>A	c.[126C>T; 110T>C; 103A>G]	rs1246774295	5′UTR	NC	[80]
C5	c.-110T>C	c.[126C>T; 113G>A; 103A>G]	rs909177624	5′UTR	NC	[80]
C5	c.-103A>G	c.[126C>T; 110T>C; 113G>A]	rs573835051	5′UTR	NC	[80]
C5	c.1A>C	p.(Met1Leu)	Hmo671	Exon 1	SW	[101]
C5	c.1A>G	p.(Met1Val)	Hmo670	Exon 1	SW	[101]
C5	c.2T>C	p.(Met1Thr)	CM1211226	Exon 1	SW/SV?	[102, 103]
C5	c.3G>A	p.(Met1Ile)	CM040727	Exon 1	SW	[104]
C5	c.23_32del	p.(Leu8Profs*42)		Exon1	SW	[105]
C1	c.29_31delTGC	p.(Leu10del)	rs61338903	Exon 1		[106]
C2	c.37C>A	p.(Leu13Met)	rs758864534	Exon 1	WT	[107]
C2	c.46G>A	p.(Ala16Thr)	rs63749090	Exon 1	NC/WT?	[107, 108]
C2	c.49C>T	p.(Arg17Cys)	rs757608533	Exon 1	NC/WT?	[107]
C5	c.59G>A	p.(Trp20*)	rs72552743	Exon 1	SW	[109]
C5	c.60G>A	p.(Trp20*)	rs746097144	Exon 1		[110]
C5	c.64dup	p.(Trp22Leufs*58)		Exon 1	SW	[111]
C5	c.68G>A	p.(Trp23*)	CM076139	Exon 1	SW	[112, 113]
C5	c.69G>A	p.(Trp23*)	rs72552744	Exon 1		[112]
C5	c.85dup	p.(His29Profs*51)		Exon 1	SW	[114]
C5	c.92C>A	p.(Pro31Gln)		Exon 1	SW	[115]
C5	c.92C>T	p.(Pro31Leu)	rs9378251	Exon 1	NC-SV	[116–121]
C5	c.116A>T	p.(His39Leu)	rs1030467767	Exon 1	SV ?	[122]
C5	c.124C>T	p.(Gln42*)	CM117607	Exon 1	SW	[123]
C4	c.129del	p.(Asp44Thrfs*9)	CD043179	Exon 1	SW/SV	[124]
C5	c.137_138delinsTG	p.(Pro46Leu)	CI060691	Exon 1	SW-SV	[125]
C5	c.138dupC	p.(Ile47Hisfs*33)		Exon 1	SV	[126]
C5	c.143A>G	p.(Tyr48Cys)	rs566306310	Exon 1	NC	[127]
C5	c.144delT	p.(Leu49Cysfs*4)		Exon 1	SW	[22]
C5	c.163A>T	p.(Lys55*)	CM098017	Exon 1	SW	[128]
C5	c.169G>A	p.(Gly57Arg)	CM082589	Exon 1	SV	[129]
C5	c.178T>A	p.(Tyr60Asn)	HM0672	Exon 1	SW	[101]
C5	c.188A>T	p.(His63Leu)	rs9378252	Exon 1	NC	[130]
C5	c.194G>A	p.(Gly65Glu)	CM990459	Exon 1	SW	[131]
C5	c.203-2A>G	Disrupted splice acceptor	CS961545	Intron 1	SW	[112]
C5	c.212_213insTGTGGTGGTG	p.(Leu72Valfs*11)		Exon 2	SW	[132]
C4	c.208G>T	p.(Val70Leu)	rs763599355	Exon 2	SV	[119]
C5	c.223A>T	p.(Lys75*)	CM990460	Exon 2	SW	[133]
C5	c.233T>C	p.(Ile78Thr)	CM050039	Exon 2	SV	[120, 134]
C5	c.272G>T	p.(Gly91Val)	CM990461	Exon 2	SW	[133]
C4	c.274A>G	p.(Arg92Gly)		Exon 2	SW	[119]
C5	c.274A>T	p.(Arg92*)		Exon 2	SW	[24]
C5	c.292dupT	(p.Tyr98Leufs*6)	CI138738	Exon 2	SW	[135]
C5	c.292+1G>A	Disrupted splice donor	rs779144910	Intron 2	SW	[119, 136]
C5	c.292+5G>A	disrupted donor splice site	rs757288233	Intron 2	SW	[137]
C1	c.293-79G>A		rs114414746	Intron 2		[103]
C1	c.293-13C>A		rs6467	Intron 2	WT	[65]
C5	c.293-13C>G	New splice acceptor site	rs6467	Intron 2	SW	[65, 117–119]
C3	c.293-7C>G	intron 2 acceptor splice site	rs193922544	Intron 2	SW	[138]
C5	c.293-2A>G	Disrupted splice acceptor	CS022262	Intron 2	SW	[139]
C5	c.294C>A	p.(Tyr98*)	CM980506	Exon 3	SW	[140]
C3	c.304_305delinsAA	p.(Ser102Asn)		Exon 3	NC/WT?	[107]
C1:	c.308G>A	p.(Arg103Lys)	rs6474	Exon 3	WT	[125]
C5	c.317C>T	p.(Pro106Leu)	CM940328	Exon 3	NC	[141]
C5	c.323T>G	p.(Leu108Arg)	CM082591	Exon 3	SW	[129]
C4	c.323T>A	p.(Leu108Gln)	rs957886272	Exon 3	SV-NC	[142]
C5	c.332_339del	p.(Gly111Valfs*21)	rs387906510	Exon 3	SW	[118–120, 143]
C4	c.341C>T	p.(Ser114Phe)	rs1296268275	Exon 3	SV-NC	[107, 24]
C4	c.341C>A	p.(Ser114Tyr)	rs1296268275	Exon 3	NC	[24, 144]
C5	c.359A>G	p.(His120Arg)	HM070142	Exon 3	NC	[145]
C5	c.364A>C	p.(Lys122Gln)	rs547552654	Exon 3	NC	[146]
C4	c.368T>C	p.(Leu123Pro)		Exon 3	SW	[147]
C4	c.368T>G	p.(Leu123Arg)		Exon 3	NC	[142]
C4	c.371C>T	p.(Thr124Ile)	rs566065375	Exon 3	SW	[119]
C5	c.373C>T	p.(Arg125Cys)	rs371412889	Exon 3	SV-NC	[148]
C5	c.374G>A	p.(Arg125His)	rs72552750	Exon 3	NC	[104]
C4	c.389T>C	p.(Leu130Pro)		Exon 3	SW	[118]
C3	c.397C>T	p.(Arg133Cys)	rs770379536	Exon 3	NC	[149, 150]
C3	c.398G>A	p.(Arg133His)		Exon 3	NC	[142]
C5	c.419T>A	p.(Val140Glu)	CM122724	Exon 3	SW	[151]
C5	c.421G>A	p.(Glu141Lys)	rs774422392	Exon 3	SW	[125, 152]
C4	c.424C>T	p.(Gln142*)	CM130871	Exon 3	SW	[148]
C5	c.428T>C	p.(Leu143Pro)	CM082590	Exon 3	SW	[129]
C4	c.434A>C	p.(Gln145Pro)		Exon 3	SW	[119]
C5	c.442T>C	p.(Cys148Arg)	CM122725	Exon 3	SV-NC	[125, 152]
C5	c.447+1G>A	Disrupted splice donor		Intron 3	SW	[153]
C5	c.448C>T	p.(Arg150Cys)	rs577450124	Exon 4	NC	[149, 150]
C3	c.449G>C	p.(Arg150Pro)	rs760710835	Exon 4	NC	[154]
C5	c.452T>G	p.(Met151Arg)	CM115995	Exon 4	SV-NC?	[147]
C5	c.460C>T	p.(Gln154*)	rs775389993	Exon 4	SW	[119]
C5	c.481dupA	p.(Ile161Asnfs*)		Exon 4	SW	[155]
C2	c.478G>A	p.(Ala160Thr)	rs761406994	Exon 4	WT	[125]
C4	c.484G>T	p.(Glu162*)		Exon 4	SW	[147]
C5	c.492delA	p.(Glu164Aspfs*)		Exon 4	SW	[156]
C5	c.494T>C	p.(Phe165Ser)		Exon 4	CL-SV	[119]
C3	c.496T>C	p.(Ser166Pro)		Exon 4	NC	[118]
C5	c.500T>C	p.(Leu167Pro)	CM071684	Exon 4	SW	[157]
C5	c.503T>C	p.(Leu168Pro)	CM101300	Exon 4	SW	[158]
C4	c.506C>A	p.(Thr169Asn)	CM106846	Exon 4	NC	[117]
C5	c.508T>C	p.(Cys170Arg)	CM066041	Exon 4	SV	[159]
C5	c.509_510insA	p.(Cys170*)		Exon 4	SW	[160]
C5	c.510C>A	p.(Cys170*)		Exon 4	SW	[117]
C4	c.511dup	p.(Ser171Lysfs*125)	rs1378695952	Exon 4	SW	[139]
C5	c.515T>A	p.(Ile172Asn)	CM062571	Exon 4	SV	[161]
C5	c.518T>A	p.(Ile173Asn)	rs6475	Exon 4	SV	[107, 117–120, 125, 162]
C5	c.535G>A	p.(Gly179Arg)	rs772317717	Exon 4	SW	[159]
C5	c.536G>C	p.(Gly179Ala)	rs72552751	Exon 4	SV	[133]
C5	c.549+1G>C	Disrupted splice donor		Intron 4	SW	[119]
C5	c.550-1G>A	Disrupted splice acceptor		Intron 4	SW	[163]
C5	c.552delC	p.(Asp184Glufs*)		Exon 5	SW	[164]
C1	c.552C>G	p.(Asp184Glu)	rs397515531	Exon 5	WT	[165, 166]
C5	c.574T>C	p.(Tyr192His)	CM119139	Exon 5	NC	[167]
C5	c.584T>A	p.(Ile195Asn)	HM070141	Exon 5	NC	[128]
C3	c.590_592delAGG	p.(Glu197del)		Exon 5	NC, SV?	[168]
C5	c.597A>T	p.(Leu199Phe)	rs143240527	Exon 5	NC?	[169]
C3	c.607A>G	p.(Ser203Gly)	rs372964292	Exon 5	NC-WT	[107]
C5	c.634G>A	p.(Val212Met)	rs758846970	Exon 5	WT ?	[125, 103]
C5	c.634G>C	p.(Val212Leu)	CM880021	Exon 5	ndea	[158, 170]
C4	c.639dupT	p.(Pro214Serfs*)		Exon 5	SW	[104]
C5	c.652-8T>A	Disrupted splice acceptor		Intron 5	SW, SV?	[163]
C5	c.652-2A>G	Disrupted splice acceptor	rs372403269	Intron 5	SW	[149]
C4	c.662del	p.(Asn221fs*)	CD138173	Exon 6	SW	[171]
C5	c.673C>T	p.(Arg225Trp)	HM070087	Exon 6	NC	[172]
C4	c.676_677del	p.(Arg226fs*)		Exon 6	SW	[24]
C4	c.683dup	p.(Gln229Alafs*67)		Exon 6	SW	[173]
C5	c.685C>T	p.(Gln229*)	rs72552752	Exon 6	SW	[174]
C5	c.692T>C	p.(Ile231Thr)	CM101304	Exon 6	NC	[158]
C5	c.700A>G	p.(Arg234Gly)	CM081569	Exon 6	NC/SV?	[121, 175]
C5	c.701G>A	p.(Arg234Lys)	CM105548	Exon 6	SV	[158]
C1	c.705T>C	p.(Asp235=)	rs10947229	Exon 6		[165]
C5	c.710T>A	p.(Ile237Asn)	rs111647200	Exon 6	SV	[119, 120, 176]
C5	c.713T>A	p.(Val238Glu)	rs12530380	Exon 6	SW	[119, 120, 176]
C5	c.715G>A	p.(Glu239Lys)	rs754019944	Exon 6	ndea	[103]
C4	c.715_717delGAG	p.(Glu238del)		Exon 6	SW	[177]
C5	c.719T>A	p.(Met240Lys)	rs6476	Exon 6	WT	[119, 120, 176]
C4	c.740del	p.(Glu247Glyfs*)	CD021411	Exon 7	SW	[119]
C5	c.749T>C	p.(Val250Ala)	rs200778936	Exon 7	NC	[178]
C5	c.785T>C	p.(Leu262Pro)	rs750337015	Exon 7	SW	[179]
C5	c.787C>T	p.(Gln263*)	CM990463	Exon 7	SW	[131]
C4	c.787dup	p.(Gln263Profs*)	CM990463	Exon 7	SW	[120]
C3	c.790G>C	p.(Gly264Arg)		Exon 7		[67]
C2	c.796G>T	p.(Ala266Ser)		Exon 7	WT	[121]
C5	c.797C>T	p.(Ala266Val)	rs144029176	Exon 7	NC	[180]
C1	c.803C>T	p.(Pro268Leu)	rs61732108	Exon 7	WT	[107]
C1	c.806G>C	p.(Ser269Thr)	rs6472	Exon 7	WT	[165, 181, 182]
C5	c.844G>C	p.(Val282Leu)	rs6471	Exon 7	NC	[118, 183]
C5	c.844G>T	p.(Val282Leu)	rs6471	Exon 7	NC	[107, 117, 119–121, 184]
C5	c.845T>G	p.(Val282Gly)	CM000364	Exon 7	SV-NC	[22]
C5	c.847C>A	p.(His283Asn)	CM119136	Exon 7	SV	[167]
C5	c.850A>G	p.(Met284Val)	rs770199817	Exon 7	NC	[149, 150]
C5	c.850A>T	p.(Met284Leu)	CM023732	Exon 7	NC	[185]
C5	c.874G>A	p.(Gly292Ser)	rs201552310	Exon 7	SW	[141, 164, 168]
C5	c.874G>C	p.(Gly292Arg)	rs201552310	Exon 7	SW	[125, 151, 164]
C5	c.874G>T	p.(Gly292Cys)	CM990464	Exon 7	SW	[133]
C5	c.877G>A	p.(Gly293Ser)	rs151344501	Exon 7	SV/SW?	[186]
C5	c.878G>A	p.(Gly293Asp)	CM101301	Exon 7	SW	[158]
C5	c.887C>A	p.(Thr296Asn)	CM122726	Exon 7	SW-SV	[151]
C5	c.901C>T	p.(Leu301Phe)	CM000365	Exon 7	SV	[22]
C5	c.905C>A	p.(Ser302Tyr)	CM031956	Exon 7	NC	[164]
C5	c.907T>C	p.(Trp303Arg)	CM066042	Exon 7	SW	[159]
C5	c.908G>C	p.(Trp303Ser)	CM060249	Exon 7	SV	[126, 187]
C5	c.909G>A	p.(Trp303*)	rs777168794	Exon 7	SW	[188]
C5	c.913G>A	p.(Val305Met)	rs151344505	Exon 7	NC	[189]
C5	c.914T>A	p.(Val305Glu)		Exon 7	SW	[119]
C3	c.917T>C	p.(Val306Ala)	rs568758408	Exon 7	SV	[144]
C4	c.919T>G	p.(Phe307Val)	rs746303150	Exon 7	NC	[144, 165]
C4	c.921T>G	p.(Leu308Val)		Exon 7	NC	[24, 144]
C5	c.923dup	p.(Leu308Phefs*6)	rs267606756	Exon 7	SW	[118–120]
C5	c.925C>T	p.(Leu309Phe)	CM122727	Exon 7	SV	[151]
C5	c.939+1G>C	Disrupted splice donor		Intron 7	SW	[190]
C5	c.939+2T>G	Disrupted splice donor	rs72552753	Intron 7	SW	[191]
C5	c.946C>T	p.(Gln316*)	CM053199	Exon 8	SV	[192]
C5	c.949C>T	p.(Arg317*)	rs748290896	Exon 8	SW	[193]
C3	c.950G>T	p.(Arg317Leu)		Exon 8	SV-NC	[24, 144]
C5	c.952C>A	p.(Leu318Met)	CM010203	Exon 8	NC	[194]
C3	c.952C>G	p.(Leu318Val)	CM053827	Exon 8	NC	[195]
C5	c.955C>T	p.(Gln319*)	rs7755898	Exon 8	SW	[118–120, 196]
C5	c.961G>A	p.(Glu321Lys)	CM101302	Exon 8	SV	[158]
C4	c.965T>C	p.(Leu322Pro)		Exon 8	SW	[144]
C5	c.968A>G	p.(Asp323Gly)	CM060248	Exon 8	NC	[126]
C5	c.991_1000del	p.(Ser331Glyfs*)		Exon 8	SW	[193]
C5	c.1003del	p.(Val335Serfs*)	CD130873	Exon 8	SW	[148]
C3	c.1007C>T	p.(Pro336Leu)	COSM3624998	Exon 8	NC	[142]
C5	c.1011C>G	p.(Tyr337*)	rs139392370	Exon 8	NC-SW	[197]
C5	c.1019G>A	p.(Arg340His)	rs72552754	Exon 8	NC	[198]
C5	c.1024C>T	p.(Arg342Trp)	rs777860817	Exon 8	NC-SV	[121]
C5	c.1025G>C	p.(Arg342Pro)	CM033605	Exon 8	SV	[199]
C5	c.1037T>C	p.(Leu346Pro)	CM138736	Exon 8		[135]
C5	c.1054G>A	p.(Glu352Lys)	rs771822460	Exon 8	SV	[144, 200]
C4	c.1055A>T	p.(Glu352Val)		Exon 8	SW-SV	[186]
C5	c.1061T>G	p.(Leu354Arg)	CM087502	Exon 8	SW	[201]
C5	c.1063C>T	p.(Arg355Cys)	rs772900496	Exon 8	SW	[22]
C5	c.1064G>A	p.(Arg355His)	rs760216630	Exon 8	SW	[133]
C5	c.1064G>C	p.(Arg354Pro)		Exon 8	SW	[202]
C5	c.1069C>T	p.(Arg357Trp)	rs7769409	Exon 8	SW	[118–120, 147, 203]
C5	c.1070G>A	p.(Arg357Gln)	rs574370139	Exon 8	SV	[119, 204]
C5	c.1070G>C	p.(Arg357Pro)	CM970414	Exon 8	SW	[165, 205]
C4	c.1075G>A	p.(Val359Ile)	rs373579128	Exon 8		[206]
C5	c.1088C>T	p.(Ala363Val)	CM990466	Exon 8	SW	[131]
C5	c.1091T>G	p.(Leu364Trp)	CM013257	Exon 8	SV	[207]
C5	c.1096C>T	p.(His366Tyr)	CM042969	Exon 8	SW	[120, 124]
C4	c.1096C>A	p.(His366Asn)		Exon 8	NC	[165]
C2	c.1099C>T	p.(Arg367Cys)	rs758658540	Exon 8	NC	[121]
C3	c.1100G>A	p.(Arg367His)	rs376035565	Exon 8	NC	[144]
C5	c.1108C>T	p.(Arg370Trp)	rs781074931	Exon 8	NC	[158]
C5	c.1118+1G>A	Disrupted splice donor	rs778895502	Intron 8	SW	[120]
C5	c.1119-2A>G	Disrupted splice acceptor	rs1256824831	Intron 8	SW	[163]
C5	c.1126G>A	p.(Gly376Ser)	rs151344506	Exon 9	SW	[189]
C5	c.1131C>A	p.(Tyr377*)	CM031957	Exon 9	SW	[164]
C5	c.1143G>C	p.(Glu381Asp)	rs72552756	Exon 9	SW	[208]
C4	c.1144G>A	p.(Gly382Ser)	rs1395322291	Exon 9	SW	[144]
C4	c.1160C>T	p.(Pro387Leu)	rs546660952	Exon 9	NC	[144]
C4	c.1160C>G	p.(Pro387Arg)		Exon 9	SW	[117]
C5	c.1164C>G	p.(Asn388Lys)	CM099830	Exon 9	NC	[209]
C5	c.1166T>G	p.(Leu389Arg)	CM128445	Exon 9	SW	[125, 152]
C4	c.1170_1178del	p.(Gln390_Ala392del)		Exon 9	SW	[107]
C5	c.1174G>A	p.(Ala392Thr)	rs202242769	Exon 9	NC	[157]
C5	c.1179_1194dup16	p.(Trp399Profs*)		Exon 9	SW	[136]
C4	c.1213T>C	p.(Phe405Leu)		Exon 9	SW	[24]
C5	c.1214T>C	p.(Phe405Ser)	CM074139	Exon 9	SW	[210]
	c.1215C>A	p.(Phe405Leu)		Exon 9	SW/NC?	[24, 144]
C5	c.1217G>A	p.(Trp406*)	rs151344503	Exon 9	SW	[120, 190]
C5	c.1222G>A	p.(Asp408Asn)	HM070140	Exon 9	NC	[172]
C4/C5	c.1222+1G>C	Disrupted splice donor		Intron 9	SW	[148]
C4	c.1223-9C>A	New abberr. splice acc.	rs748777524	Intron 9	SW	[211]
C4	c.1223-1G>A	Disrupted splice acceptor	CS110243	Intron 9	SW	[120]
C5	c.1225C>T	p.(Arg409Cys)	rs72552757	Exon 10	SW	[120]
C4	c.1226G>A	p.(Arg409His)	rs1351045983	Exon 10	SW-SV	[120, 144]
C5	c.1226G>A	p.(Arg409His)	CM110245	Exon 10	SV	[120]
	c.1226G>T	p.(Arg409Leu)		Exon 10	SW	[212]
C4	c.1273_1277del	p.(Gly425Profs*97)		Exon 10	SW	[120]
C5	c.1273G>A	p.(Gly425Ser)	rs72552758	Exon 10	SV	[213]
C5	c.1279C>T	p.(Arg427Cys)	CM066039	Exon 10	SW	[159]
C5	c.1280G>C	p.(Arg427Pro)	CM110246	Exon 10	NC/SV	[120, 144]
C5	c.1280G>A	p.(Arg427His)	rs151344504	Exon 10	SW	[214]
C4	c.1285T>C	p.(Cys429Arg)		Exon 10	SW	[119]
C4	c.1291_1292del	p.(Gly431Argfs*)		Exon 10	SW	[150]
C5	c.1294G>A	p.(Glu432Lys)	HM060572	Exon 10	NC	[149, 215]
C5	c.1298C>T	p.(Pro433Leu)	rs751456004	Exon 10	NC-SV	[216]
C4	c.1301T>C	p.(Leu434Pro)	rs1228433585	Exon 10		[144]
C4	c.1301T>C	p.(Leu434Pro)	rs1228433585	Exon 10	SW	[24]
C5	c.1304C>T	p.(Ala435Val)	CM050040	Exon 10	SV	[134]
C4	c.1304C>A	p.(Ala435Glu)		Exon 10	SW	[216]
C5	c.1306C>T	p.(Arg436Cys)	rs767333157	Exon 10	NC	[194]
C5	c.1333C>T	p.(Arg445*)	CM060247	Exon 10	SW	[120, 126]
C4	c.1334G>C	p.(Arg445Pro)	rs1465580356	Exon 10	SW	[144]
C5	c.1340T>C	p.(Leu447Pro)	CM062572	Exon 10	SW	[161]
C5	c.1351A>C	p.(Thr451Pro)	CM074138	Exon 10	SW	[107, 210]
C3	c.1352C>T	p.(Thr451Met)	rs1319651744	Exon 10	mild NC	[107]
C5	c.1360C>T	p.(Pro454Ser)	rs6445	Exon 10	NC	[107, 118, 121, 217]
C5	c.1378C>T	p.(Pro460Ser)	CM106849	Exon 10	SV	[117]
C5	c.1379C>A	p.(Pro460His)	CM078111	Exon 10	NC	[218]
C4	c.1379C>T	p.(Pro460Leu)	CM078111	Exon 10	SW-SV	[119]
C3	c.1381_1398del	p.(Ser461_Pro466del)		Exon 10	NC	[110]
C5	c.1391C>T	p.(Pro464Leu)	CM060246	Exon 10	SV	[126]
C4	c.1393del	p.(Leu465Cysfs*)		Exon 10	SW	[119]
C4	c.1399dupC	p.(His467Profs*)		Exon 10	SW	[142]
C3	c.1420_1440dup	p.(Met474_Arg480dup)		Exon 10	SV	[126]
C3	c.1422G>T	p.(Met474Ile)	rs1312209092	Exon 10	NC	[121]
C4	c.1430del	p.(Phe477Serfs*65)		Exon 10	SW	[219]
C5	c.1439G>T	p.(Arg480Leu)	rs184649564	Exon 10	NC	[124]
C5	c.1444C>T	p.(Gln482*)		Exon 10	SW	[147]
C5	c.1445A>C	p.(Gln482Pro)	CM056573	Exon 10	SW	[220]
C5	c.1447C>T	p.(Pro483Ser)	rs776989258	Exon 10	NC	[107, 120, 121, 221]
C5	c.1450C>T	p.(Arg484Trp)	rs759736443	Exon 10	SW	[109, 119]
C4	c.1451G>C	p.(Arg484Pro)	rs200005406	Exon 10	SV	[119, 120, 148, 168]
C5	c.1451G>A	p.(Arg484Gln)	rs200005406	Exon 10	SV	[164]
C5	c.1451G>C	p.(Arg484Pro)	rs200005406	Exon 10		[119, 120, 222]
C4	c.1454_1455del	p.(Gly485Aspfs*)		Exon 10		[119]
C5	c.1455del	p.(Met486Trpfs*56)	rs749280425	Exon 10		[119, 190, 223]
C1	c.1481G>A	p.(Asn493Ser)	rs6473	Exon 10	ndea	[106, 181]
C4	c.1483dup	p.(Gln495Profs*)		Exon 10	SW	[103]
C1	c.*12C>T		rs150697472	3′UTR	NC	[224]
C3	c.*13G>A		rs6447	3′UTR	NC	[206]
C1	c.*52C>T		rs1058152	3′UTR	NC	[224]
C1	c.*440C>T			3′UTR	NC	[224]
C1	c.*443T>C			3′UTR	NC	[224]

*ndea* no detectable enzyme activity

The curated list of variations was used as input for an automated database search of known variations listed in release 97 of the Ensembl database1 to complement and proofread information [227]. Remaining discrepancies that could not be resolved automatically were highlighted and resolved by hand.

Large deletions and conversions extending to about 30kb with breakpoints between exons 3 and 8 of *CYP21A1P* through C4B to the corresponding point in *CYP21A2* comprise about 20–30% of CAH alleles [1, 2, 69]. Only a small number of variants affecting 21-OH function (~10%) represent new variants not derived from the pseudogene. CYP21A2 de novo germ-line variantss are estimated to account for about 1–2% of CAH alleles in unexpectedly affected newborns [1, 2, 70]. In case that only one parent of a child (suffering from CAH) carries a disease causing variant, it can be deduced that the child’s second allele has harbored the disease causing variant “de novo”. That means that for children from further pregnancies of that couple there is no significantly increased risk to t should be mentioned that it is still a matter of dbe affected by CAH.

The close proximity of the functional *CYP21A2* gene to the pseudogene, as well as copy number variations of the RCCX unit, have been assumed to predispose to de novo gene deletions due to unequal crossing-over between chromosomes with a monomodular and a standard bimodular form (compare Fig. 2) [66, 70], the latter including two complete functional copies of the *CYP21A2* gene. It has been suggested that such a bimodular chromosome has no equally-sized homologue to align to during meiosis, resulting in misalignment of a *CYP21A1P* pseudogene to the functional *CYP21A2* gene and causing a deletion of a 30 kb region including the *CYP21A2* gene and the 3′ portion of the *TNXB* gene.

In that context it is of note that in spite of the detection of two *CYP21A2* copies, this could be due to a duplicaton of *CYP21A2* on one allele, whereas the second allele is lacking a functional *CYP21A2* gene, resulting in a CAH-carrier state.

#### Genotype-phenotype correlation

In general there is a good genotype/phenotype correlation [22, 23] and specific genotypes have been shown to be associated with salt wasting, simple virilizing or non-classical CAH. The genotype/phenotype correlation decreases with diminished severity of the disease [71, 72] or depends on the patient’s background with respect to other genes regulating androgen and oestrogen metabolism. In that context it is of note that Grodnitskaya and Kurtser [73] published only recently that out of 800 women with hyperandrogenism only 1% had non-classical CAH due to 21-OH deficiency. There are also many papers reporting symptomatic patients with clinical signs of hyperandrogenism (hirsutism, precocious puberty, cycle abnormalities in women) and only heterozygous 21-OH function impairing variants and that heterozygotes are more likely to have signs of androgen excess than would genetically-unaffected subjects [74–76]. Nevertheless, a recent study [77] showed that out of 205 patients with hyperandrogenemia manifested in adolescence or adulthood the majority (*n* = 105) were not carriers of 21-OH function impairing variants. Moreover, due to our experience, almost all (heterozygous) parents of classical CAH-patients were clinically asymptomatic or unaware of CAH-related symptoms and were thus identified as 21-OHD-carriers only in the course of family analysis of a 21-OHD index patient. At present it is unclear which conditions lead to “mild” clinical symptoms in heterozygous carriers. As addressed previously [77], benign variants of *CYP21A2* (as p.(Asn493Ser) c.1478G>A) could influence the phenotype. Other defects (at a genetic or environmental level) in adrenal and ovarian steroid metabolism and/or metabolic disturbances (e.g., hyperinsulinism, obesity) should be investigated as possible causes for the hyperandrogenism. In these patients, detection of disease causing *CYP21A2* variants is only important in the background of family planning.

Another reason for genotype-phenotype variability is the so-called leakiness (e.g., c.290-13C>G) or a different molecular “background” of the respective variant (e.g., p.(Pro30Leu)—small versus large gene conversion – or the circumstance that 3′-and 5′-UTR (untranslated region) are often not analyzed in the course of routine *CYP21A2* genotyping [25, 26, 69, 78–82]. This has to be kept in mind, when interpreting and reporting results of 21-OH genotyping.

## Methodological approaches—analytical methods

### Best practice 21-OHD genotyping

Due to the complex spectrum of disease causing variants (large gene conversions with multiple mutations in cis, deletions and duplications of variable size [83–89], more than one 21-OH function affecting variant per gene), *best practice genotyping* should be *PCR-based sequence analysis along with MLPA*, which would detect the majority of types of potential alterations.

If other methodologies are used, which cover only the most common disease causing variants and/or cannot detect large deletions/duplications the limitations of the employed methods have to be clearly stated in the respective genetic reports.

Nevertheless, we have to be aware, that all methods mentioned so far have limitations and none of those techniques are able to identify 100% of possible variants due to the complexity of the *CYP21A2* locus.

In at-risk relatives despite known familial *CYP21A2* variants a full screening for *CYP21A2* variants (by sequence and MLPA analysis) is preferable, rather than exclusively analyzing the known 21-OH function impairing variant detected in the index patient. Due to a high *CYP21A2*-carrier rate (as addressed previously in these guidelines [21, 42]) family members may carry other *CYP21A2* variants than detected in the index patient and would then escape detection.

### Approved/preferred methodologies

#### PCR-based sequence analysis

Usually PCR-based Sanger sequencing is performed to detect disease causing or clinically relevant single nucleotide substitutions, small deletions and insertions. In brief, one or more fragments covering all 10 exons and the respective exon/intron boundaries are specifically amplified by selective PCR primers differentiating the functional *CYP21A2* gene from the *CYP21A1P* pseudogene followed by Sanger sequencing. So far, only the minority of laboratories sequence parts of the 5′ regulatory and of the 3′ untranslated region.

Of note, PCR based approaches detecting sequence variants, the common 30kb deletion as well as fusion events can also be used for comprehensive *CYP21A2* genotyping [89].

##### Primer design

Since exonic and intronic nucleotide sequences of the pseudogene and the functional gene exhibit 98% sequence homology [61] and are characterized by a high number of SNPs, the design of PCR- and sequencing primers is challenging. A shortlist of different primers used for PCR-based sequencing is provided as Supplementary material.

In principle, allelic dropout has to be considered as for every other gene, since SNPs could lie in primer regions and hamper primer binding. Sequencing overlapping fragments with different primers can help to detect allelic drop out and hence minimize the probability of missing disease causing variants.

##### Taq-polymerase

The use of proof reading Taq-polymerases is strongly recommended to avoid allelic drop out, as previously reported for the c.290-13C>G locus [90]. Laboratory experience suggests that this phenomenon does not particularly relate to this intronic locus but could be a problem also for other loci.

##### Promoter and 3′ UTR

Whereas exons and intron/exon boundaries are usually covered by PCR-based sequencing, the majority of the laboratories do not analyze the promoter and the 3′ untranslated region in the course of routine *CYP21A2* genotyping. As reported previously [79–82] promoter variants could result in classic as well as non-classic CAH and/or could modify the phenotype in patients with p.(Pro30Leu) variants [69, 78]. However, due to that lack of information on patients’ data the clinical relevance of promoter sequence variants is unclear and interpretation therefore remains difficult.

In that context it is of note that MLPA covers the c.-113G>A variant in the promoter region with the respective probe for exon 1, but the knowledge on a deletion/conversion in exon 1 (as detected by MLPA) cannot replace for the sequencing of at least a part of the promoter region.

It was reported [26] only recently that the variant c.*13G>A correlates with a mild form of CAH. This variant should therefore be included in routine *CYP21A2* genotyping.

#### Multiplex ligation-dependent probe amplification (MLPA)

A commercially available *CYP21A2*-MLPA kit is widely used, as this method [29, 91, 92] has the advantage that it is easy to set up and that results should be comparable between laboratories worldwide, although the automatic computer-based plotting is individually done by each lab using different analysis software products. Substitutions of MLPA probes and software updates by the manufacturer require re-evaluation and re-validation of that method (including the used software) regarding IVD (in vitro diagnostic) policies, since the assay is not CE-certified and probe specificity and exons covered by the test may change. Therefore, we strongly encourage the documentation of the MLPA-lot used in the genetic report.

#### Variant-specific rapid screening strategies

Different methods allowing rapid detection of the most common variants causing CAH are performed by certain laboratories and/or certain countries in the course of stepwise analysis of 21-OH deficiency. Such methods, reviewed in more detail by others [1, 93], include allele-specific oligonucleotide hybridization, amplification-created restriction site and single-stranded conformational analysis, allele-specific oligonucleotide PCR, oligonucleotide arrays, ligase chain reaction or PCR-based minisequencing as well as a commercially available CE-IVD certified Strip assay [40], detecting the most common 21-OH function impairing variants in a time- and cost-effective manner. The latter assay can be used as an independent confirmation for already detected variants by other assays and strategies. Of note, recent EMQN-CAH- schemes revealed that several laboratories using that method did not have sufficient knowledge and experience in interpreting *CYP21A2* constellations, as they reported wrong results using that assay. In addition, it was observed by recent EMQN CAH-schemes that disease causing variants as c.898C>T p.(Leu300Phe) were missed by such “CAH-variant-specific” methods. Detection of one disease-causing variant does not exclude the presence of further ones not covered by the respective assay. This has to be mentioned in the respective genetic reports and should also be considered in genetic counseling.

#### Next generation sequencing

Massive parallel sequencing is expanding quickly and represents a promising tool for future molecular diagnostic approaches. Although already used for 21-OHD genotyping by some laboratories, due to the limited experience a recommendation cannot be given at this point of time. In the course of the 2017 EMQN-CAH scheme two laboratories used NGS and one failed to detect the very common c.515T>A p.(Ile172Asn) variant (in the heterozygous state) in one case, but detected it in the second (in homozygous form). On the basis of the submitted reports of the recent CAH-scheme the assessors strongly encourage to include more information on bioinformatics tools as well as on enrichment kit, etc. in order to get more knowledge on the NGS technique for *CYP21A2* analysis. Preliminary data (unpublished observation by the authors) demonstrate difficulties in discriminating localization of variants regarding homologous genes and pseudogenes.

#### Southern blot analysis (SBA) and quantitative PCR

Originally SBA was used to detect large gene deletions and conversions [1, 2, 93], employing at least two restriction enzymes (Taq I and Bgl II), in order to differentiate large gene deletions from conversions.

Since Southern blot analysis is time-consuming and requires high concentrations and amounts of DNA, in the majority of diagnostic laboratories SBA has been replaced by MLPA or semiquantitative and quantitative real-time PCR methods. Whereas the latter have previously been developed almost exclusively as in house products in different laboratories, in the meantime an IVD-certified kit (ViennaLab) for quantitation of *CYP21A* copies is commercially available.

#### Caveats and pitfalls

##### Controls

Positive controls (with known variants in the respective analyzed exon/intron) should be used for validation of all analyses to ensure that the technique used is appropriate. For predictive testing a close relative carrying the disease causing variant could be analyzed in parallel as internal control.

##### Duplicated *CYP21A2* genes

The wide use of MLPA led to the findings that three or even four functional *CYP21A2* genes are present in a certain number of individuals. To avoid false interpretation of positive genotyping it is of utmost importance to be aware of the high frequency of duplicated *CYP21A2* genes in association with p.(Gln318*) [28, 42, 94, 95]. In case of detection of a p.(Gln318*) variant, we strongly recommend MLPA analysis in order to clarify the *CYP21A2* gene copy number. If three or more *CYP21A2* copies are present, PCR with specific primers and/or family analysis has to be performed in order to allocate the disease causing variant to the respective single or duplicated *CYP21A2* gene [42]. If two *CYP21A2*-genes are detected, the p.(Gln318*) bearing allele represents a CAH-allele. If p.(Gln318*) is on an allele carrying a duplicated *CYP21A2*, it does not represent a CAH allele, but rather a functional normal one.

Currently, the presence of three functional *CYP21A2* genes distributed on two alleles are considered to exhibit complete normal function, although proof from the literature is lacking. The observation that the disease causing variant p.(Gln318*) associated with two *CYP21A*2 gene copies on the same allele or chromosome is more prevalent in the healthy general population than in CAH-patients is in line with the above-mentioned assumption [42, 95].

Of note, the number of functional genes do not only determine the carrier status of a person, but the presence of three or more functional genes could hamper the detection of one mutated allele out of three or four in comparison to one mutated sequence out of two.

In case of duplicated *CYP21A2* alleles the risk of no correct assignment of the mutated allele and hence wrong distribution of *CYP21A2* genes during meiosis cannot be excluded.

In that context it is of note that in spite of the detection of two *CYP21A2* copies, this could be due to a duplicaton of *CYP21A2* on one allele, whereas the second allele is lacking a functional *CYP21A2* gene, resulting in a CAH-carrier state.

The above mentioned constellations have to be documented and addressed in the respective genetic reports to provide correct genetic counseling.

##### Pitfalls and false negative results

Pitfalls and false negative results can occur during *CYP21A2* analysis and are due to

- mix up of samples during venipuncture, during DNA isolation or subsequent testing.
- sequence variations in primer binding regions which could give rise to non-amplification of a normal or disease causing variant carrying allele (“allelic dropout”) [90]; Therefore not amplified *CYP21A2* genes are not sequenced and the variant on the other allele appears homozygous instead of heterozygous.
- homozygosity/hemizygosity for a disease causing variant which has to be verified by a second method (e.g., MLPA)
- duplication of the functional *CYP21A2* gene can mask a deletion on the second allele and can complicate determination of carrier status [28, 69, 94, 95], as outlined above.
- under certain circumstances hybrid genes can be co-amplified and detected disease causing variants could erroneously be assigned to the functional gene.
- contamination of fetal with maternal material in prenatal diagnosis.
- disease causing variant lies in a gene other than the one tested (including double heterozygosity for different genetic entities, e.g., one disease causing variant in *CYP21A2* and one in *CYP11B1*).

## Interpretation and reporting

### Reporting format and relevant information

While every laboratory has its own reporting format, general guidance on reporting is available from the European Molecular Genetics Quality Network (http://www.emqn.org) on the EMQN website and relevant links including Eurogentest (a project founded in order to harmonize genetic testing across Europe -www.eurogentest.org), the Association for Clinical Genomic Science (www.acgs.uk.com) or the Swiss Society of Medical Genetics (http://sgmg.ch). Some degree of standardization is of pivotal importance for the referring clinicians and for consistency between different laboratories.

The respective recommendations concerning a genetic report and the required informations are:

- Clear and concise report
  - Individual report issued
- Patient-related information
  - Patient’s name and date of birth, sex, ethnic background
  - Reason for referral
  - Name, address of referring clinician/institution
- Specimen-related information
  - Type of specimen (EDTA-blood, DNA, chorionic villi)
  - Date of referral/arrival noted
  - Laboratory reference/code
- Methods-related information
  - Testing method and respective limitations (error probability) must be included.
  - Clinical sensitivity for full *CYP21A2* screening is >95%, analytical sensitivity for sequencing and MLPA is assumed >98%.
  - Both the clinical and analytical accuracy of the assay should be empirically established by each laboratory as part of validation/verification and actually achieved values should be included in the methods.
  - HGVS nomenclature (http://www.hgvs.org) should be used for all variants, and citing the reference sequence on the report—LRG_829t1 (http://www.lrg-sequence.org), NM_000500.9 - is pivotal.
  - Benign variants should not be reported (but can be transmitted upon request).
- Disease-related information
  - OMIM number of the disease and mode of inheritance should be given (www.ncbi.nlm.nih.gov).
  - Detected variants should be related to the different phenotypes (classical CAH, salt wasting, simply virilising, non-classical CAH) [24, 96] and potential risk for prenatal virilisation in females should be addressed.
- Patient and family-related information
  - Availability of carrier screening for relatives and partners should be addressed.
  - Necessity of family analysis should be addressed if applicable (allocation of different disease causing variants to two or more *CYP21A2* copies).
  - Risk for children to carry 21-OHD-alleles should be addressed and given.

- In case CAH-index patient is a child.
  - Parents should be genotyped in order to exclude a de novo 21-OH function impairing variant in the affected child and to correctly calculate the risk for the couple’s further children to suffer from CAH (particularly address virilisation of external genitalia in females).
  - Necessity/opportunity of genetic counseling of the patient and her/his partner at a later time point for future children should be mentioned.

- Laboratory related information
  - Name, address of laboratory and its head.
  - Report dated and signed by two suitably qualified persons.

- Recommendations
  - Recommend retesting of an independent blood sample particularly in the case of carrier testing (family planning)
  - Recommend genetic counseling for the index patient

### Genotype related informations

The *CYP21A2* gene harbors benign variants affecting the numbering of nucleotides and codons. Thus, differences in nomenclature existing in the literature and resulting from different reference sequences may cause confusion and make comparison of reports from different laboratories difficult for referring clinicians. Therefore a common reference sequence and standardized nomenclature are desirable. The authors of this manuscript recommend LRG_829t1 (based on NM_000500.9) as a reference sequence.

HGVS nomenclature should be used for all variants including single nucleotide variants, short del/ins and large deletions; for complex variants (insertion–deletions) descriptive explanations can be given in addition.

- Definitions—explanations
  - In general, the terms “mutation” and “polymorphism” should no longer be used. The term *variant* is preferred with an explanation of whether the found variants are disease-causing, benign (and so not usually reported) or of uncertain significance.
  - SNP and polymorphisms imply a population allelic frequency of above 1%, but give no indication on pathogenicity.

### Classification of variants

Variant curation and interpretation is complex and has to be performed in reference to the established guidelines from ACMG (American College of Medical Genetics and Genomics) and ACGS (ACGS Best Practice Guidelines for Variant Classification in Rare Disease 2020). In these guidelines only some important aspects can be addressed: Common variants already well known in the literature and being listed in databases e.g., as ClinVar and already classified as “disease causing or pathogenic” do not need further investigation. All other variants, as well as not so common but already included variants in databases, need always a systematic (re-) evaluation for pathogenicity. This can be done by use of so-called search tools as e.g., ClinGen Pathogenicity Calculator (http://calculator.clinicalgenome.org/site/cg-calculator) [97] which interprets the variant on the base of ACMG guidelines (American College of Medical Genetics and Genomics) [98, 99]. The classification is performed in five classes [100]. The systematic interpretation of a variant requires at least

- the check for frequency in the respective and other populations (dbSNP, 1000 Genomes, ExAC),
- the search in different databases (for example: Pubmed, HGMD, HGVS, Pharmvar—previously CYP21-alleles, etc.),
- to perform in silico analysis (for example: MutationTaster, SIFT, PolyPhen-2, SpliceSiteFinder, INNSPLICE, MaxEntScan, etc.),
- the check of co-segregation with the disease, although this is difficult in recessive disease, and
- to perform in vitro expression experiments (is not a recommendation, but would bring information about novel variants which would be of general interest).
